# An open-label observational study and meta-analysis of non-invasive vagus nerve stimulation in medically refractory chronic cluster headache

**DOI:** 10.3389/fneur.2023.1100426

**Published:** 2023-03-30

**Authors:** Lucy Simmonds, Susie Lagrata, Anker Stubberud, Sanjay Cheema, Erling Tronvik, Manjit Matharu, Salwa Kamourieh

**Affiliations:** ^1^Headache and Facial Pain Group, UCL Queen Square Institute of Neurology and National Hospital for Neurology and Neurosurgery, London, United Kingdom; ^2^Norwegian Centre for Headache Research, Department of Neuromedicine and Movement Science, NTNU Norwegian University of Science and Technology, Trondheim, Norway; ^3^Department of Neurology, St. Olavs Hospital, Trondheim, Norway

**Keywords:** chronic cluster headache, non invasive vagus nerve stimulation, medically refractory, meta-analysis, vagus nerve (VN) stimulation

## Abstract

**Background:**

Many patients with cluster headache (CH) are inadequately controlled by current treatment options. Non-invasive vagus nerve stimulation (nVNS) is reported to be effective in the management of CH though some studies suggest that it is ineffective.

**Objective:**

To assess the safety and efficacy of nVNS in chronic cluster headache (CCH) patients.

**Method:**

We prospectively analysed data from 40 patients with refractory CCH in this open-label, observational study. Patients were seen in tertiary headache clinics at the National Hospital for Neurology and Neurosurgery and trained to use nVNS as preventative therapy. Patients were reivewed at one month and then three-monthly from onset. The primary endpoint was number of patients achieving ≥50% reduction in attack frequency at 3  months. A meta-analysis of all published studies evaluating the efficacy of nVNS in CCH was also conducted. We searched MEDLINE and EMBASE for all studies investigating the use of nVNS as a preventive or adjunctive treatment for CCH with five or more participants. Combined mean difference and responder proportions with 95% confidence intervals (CI) were calculated from the included studies.

**Results:**

17/40 patients (43%) achieved ≥50% reduction in attack frequency at 3  months. There was a significant reduction in monthly attack frequency from a baseline of 124 (±67) attacks to 79 (±63) attacks in month 3 (mean difference 44.7; 95% CI 25.1 to 64.3; *p* < 0.001). In month 3, there was also a 1.2-point reduction in average severity from a baseline Verbal Rating Scale of 8/10 (95% CI 0.5 to 1.9; *p* = 0.001). Four studies, along with the present study, were deemed eligible for meta-analysis, which showed a responder proportion of 0.35 (95% CI 0.07 to 0.69, *n* = 137) and a mean reduction in headache frequency of 35.3 attacks per month (95% CI 11.0 to 59.6, *n* = 108), from a baseline of 105 (±22.7) attacks per month.

**Conclusion:**

This study highlights the potential benefit of nVNS in CCH, with significant reductions in headache frequency and severity. To better characterise the effect, randomised sham-controlled trials are needed to confirm the beneficial response of VNS reported in some, but not all, open-label studies.

## Introduction

Many patients with cluster headache (CH) respond to established medical treatments including verapamil and lithium. However, there is a significant unmet need as some fail to respond to the available treatments or do not tolerate these medications and therefore experience significant disability (1). These patients suffer mood disturbance and poorer quality of life (1). A high percentage of patients with cluster headache have suicidal ideation or attempt suicide. CH is associated with significant socioeconomic burden with 80% of patients reporting daily activity restriction (2–4). The most severely affected patients suffer with chronic cluster headache (CCH) which is defined as CH attacks occurring for at least a year, without remission, or with remission lasting less than 3 months. Novel treatment options are therefore warranted.

With limited new prophylactic management options in CH, targeted central and peripheral neuromodulation offers an alternative to those refractory to other treatments (5). Invasive vagal nerve stimulation has been used in patients with intractable epilepsy and depression for several decades now. In the early 2000s, an incidental improvement in co-existing migraine and cluster headache was seen in some of these patients (6, 7). This led to the development of a portable transcutaneous device stimulating the cervical portion of the vagus nerve (gammaCore^®^, electroCore LLC, Basking Ridge, NJ). The gammaCore^®^ device has been used to treat CH and migraine since 2013, and was approved for the treatment of CH in the United Kingdom in 2019 (8). However, at present in the United Kingdom, it can only be prescribed on the NHS by a headache specialist, within specific funding regulations. Use can only be continued beyond three months if the patient meets specific criteria for a reduction in their headache burden. For a patient to purchase a device without a prescription, can cost approximately £3,000 a year (9). However, it is estimated that adding gammaCore® to standard therapy can result in a cost saving of £450 for each patient due to a reduction in triptan use (8).

Several studies investigating nVNS use in CCH have identified symptom improvement (10–12) and cost effectiveness (13, 14) although one study found no effect (15). Additional studies are needed to verify its role in preventative therapy for patients with CCH, particularly those who are refractory to pharmacological treatment (16, 17). In contrast to implantable stimulators, transcutaneous devices are well tolerated and are a novel approach to treating refractory headache conditions, worthy of further investigation. Our tertiary referral centre assesses patients with chronic headaches, many being highly disabled and refractory to treatment. This facilitated our study of a large number of patients with CCH in a single centre. We hypothesised that nVNS (gammaCore^®^) would be an effective and safe preventative treatment in medically refractory CCH.

The National Institute for Health and Care Excellence (NICE) guidance encourages research and audit in the use of non-invasive neurostimulation in the management of CH (8) and several observational studies and audits of varying sizes and from different groups have been published. Therefore, we aim to highlight the available studies and provide a summary of the evidence for the effectiveness of nVNS as a preventative treatment in CCH through a systematic review and meta-analysis of published studies and the present study. Additionally, this will help identify areas of preventative neuromodulatory treatments for CCH which need further development.

## Methods

### Open-label trial

#### Study design and setting

In this prospective, open label study, patients with refractory CCH seen at the National Hospital of Neurology and Neurosurgery, were approached and offered nVNS between January 2015 and January 2018.

After a baseline period of a minimum of 1 month, where no medication changes were made, patients were trained to use the device (as detailed in the Stimulation Paradigm below) by the headache nurse. Treatment with nVNS was given for a minimum period of 3 months. During this time, patients were reviewed at 1 month and then 3 monthly from the onset of use to record their progress and review their device technique. Data was collected from the baseline period, the 3 month follow up visit and the final follow up visit. No medication changes were made during the month-long baseline period prior to device use or during the first 3 months of device use.

#### Stimulation paradigm

Patients were taught to position the gammaCore® device to the neck, over the carotid pulse, anterior to the sternocleidomastoid muscle, in a parallel position to the trachea. A preventative treatment schedule was followed: each treatment dose was 2 min in duration and patients were instructed to administer three consecutive doses twice daily. The device was not used for treatment of an acute attack.

The nVNS (gammaCore^®^) has a proprietary low-voltage electrical signal and a predetermined peak voltage and peak output current. However, patients can adjust the stimulation intensity depending on comfort. The patients in this study were asked to increase the stimulation until the point that they felt the ‘lip pull’ to ensure optimal stimulation.

#### Participants

Forty consecutive patients with refractory chronic cluster headache were identified by a headache specialist in clinic. Chronic cluster headache was defined by ICHD-3 beta criteria (18) and following the publication of the ICHD-3 criteria in 2018, we subsequently ensured that all patients fulfilled these criteria (19). The consensus European Headache Federation criteria were used to define refractory CCH, with at least three severe attacks each week despite three consecutive trials of adequate preventative treatments (20).

#### Outcome measures

The primary outcome measure was a reduction in mean monthly attack frequency of ≥50% at 3 months (weeks 9–12) compared to baseline. Secondary outcome measures were: reduction in attack frequency, attack severity, duration, headache-related disability scores (HIT-6), and usage of triptans and oxygen. Safety and tolerability were assessed during the follow-up appointments for all patients, and they were able to contact our team *via* telephone or email should they experience adverse reactions or any concerns between follow-up appointments.

#### Data sources

Prospective paper-pencil headache diaries were kept for at least 1 month at baseline and throughout follow up. Patients were asked to record every cluster attack (frequency), severity on a verbal rating scale (VRS; 0 = no pain to 10 = excruciating pain), duration of cluster headaches (minutes), and use of acute treatments including sumatriptan and oxygen.

Patients completed the Headache Impact Test-6 (HIT-6) in the baseline period, at 3 months and at final follow up.

Anonymised data was collated and analysed using Microsoft Excel and kept within password protected files. This is the primary analysis of the collected data.

#### Statistical methods

No *a-priori* power analysis was made as the treatment was initiated as a “humanitarian intervention,” and the potential number of participants was uncertain. All 40 patients were included in the analyses. Last observation carried forward was used for dropouts and in the case of missing data. Data is presented as mean ± standard deviation (SD) as well as frequencies and medians with interquartile ranges. Data was assessed for normality by visual inspection of histograms. Paired t-tests were used to analyse for systematic changes in outcomes and were reported with mean differences (MD) with 95% confidence intervals (CI). All statistical tests were two-sided with a significance level of 0.05. Bonferroni correction was used to control the Type I error inflation as a result of multiple testing of the six pre-specified outcomes. Threshold for significance after Bonferroni correction was 0.008 (0.05/6).

#### Ethical approval and consent

Treatment with nVNS was offered as a “humanitarian intervention.” Ethics board approval for the collection and publication of data was granted by Northwick Park Hospital Research Ethics Committee, London, United Kingdom (reference number: 11/LO/1709). Informed consent was obtained from all patients.

### Systematic review and meta-analysis

#### Eligibility criteria for studies

We conducted a meta-analysis of published studies on the preventative nVNS treatment of CCH and combined these with the findings of the present study. Because we expected to find few studies, whereof many observational and open-label, studies included in the meta-analysis were not required to be comparative trials. All studies, both with and without comparison groups (case series, observational studies, audits, randomised controlled trials) investigating the use of nVNS as a preventive or adjunctive treatment for CCH were considered eligible. Studies were required to include five or more participants with CCH based on IHS diagnostic criteria. We primarily sought our primary outcome of reduction in headache attack frequency and number of patients considered responders. Treatment response was considered as > = 50% reduction in headache frequency, but in cases where other definitions were used this was registered and data converted wherever possible. Articles in languages other than English, conference abstracts and unpublished studies were not considered.

#### Search strategy and data collection

MEDLINE and EMBASE was searched from its inception to 7 December 2022 with the following strategy in PubMed and Ovid respectively: “cluster headache” AND (“vagus nerve stimulation” OR “vagal nerve stimulation” OR “transcutaneous vagus nerve stimulation” OR “gammaCore”). We identified and selected studies according to PRISMA and Cochrane guidelines. Firstly, duplicates were removed using the software tool PICO Portal (New York, NY United States. Available at www.picoportal.org). Titles and abstracts from the database search were then screened for eligibility by two of the authors, working independently (AS, LS). There were no discrepancies between authors. In addition, AS hand-searched the reference lists of all encountered reviews on the topic. In cases where this was insufficient to determine study eligibility, full texts were retrieved and reviewed. Details on study methods, participants, interventions and outcomes were extracted from the eligible records and entered into data collection sheets (see Supplementary material for an overview of which data were extracted for each study). Study authors were not contacted to inquire missing data in the reports. The review was not registered in a database.

#### Data synthesis

Data synthesis and analyses were based on aggregated data. We extracted data from tables and figures and converted precision and variance data where appropriate. We calculated precision and variance statistics in-house from raw data and individual patient data provided in the papers where necessary. Frequencies were converted to 28-day periods and we used data from last follow-up. We calculated mean differences and responder proportions with 95% confidence intervals (CI) from baseline to final follow-up using an inverse variance random-effects model. Heterogeneity was assessed using the *I*^2^-statistic. All meta-analyses were made using R 3.6.0 (The R Foundation for Statistical Computing) with the open-source package *meta* v.4.11–0. No sensitivity or subgroup analysis was planned, and none were conducted. Characteristics of all included studies were summarised with a description of methods, design, participants, interventions, and outcomes. This can be found in the Supplementary material.

#### Risk of bias assessment

Risk of bias assessment was made by one of the authors using the categories of bias defined in the ROBINS-I tool (21). No assessment of risk of bias across studies (e.g., publication bias) or certainty assessment was made. The meta-analysis was not registered *a-priori*, and no specific protocol for the meta-analysis was made. Extracted data, data used in the analyses and analytic code is available from the authors upon reasonable request.

## Results

Baseline demographics and clinical characteristics are listed in Table 1. Forty consecutive patients (18 women, 22 men) with CCH and a mean age of 52 ± 13 years were prescribed nVNS for at least 3 months. Sixteen patients were simultaneously on other preventive medications for cluster headache.

**Table 1:** Patient demographics.

*Age*	
Mean (±SD)	52 (±13)
Median (Range, IQR)	52 (28–75, 44–62)
*Gender n (%)*	
Male	22 (55%)
Female	18 (45%)
*Duration Chronic phase*	
Mean (±SD)	11 years (±6)
Median (Range, IQR)	9 years (3–26, 7–14)
*Acute treatments tried: n (%)*	
Sumatriptan injection	31 (77%)
High dose and flow rate Oxygen	39 (97%)
*Number of oral preventives treatments tried*	
Mean (±SD)	7 (±2)
Median (Range; IQR)	7 (4–10; 5–8)
*Interventional and Infusion treatments n (%)*	
Greater occipital nerve blocks	36 (90%)
Responders	19 (53%)
Dihydroergotamine infusion	18 (45%)
Lidocaine infusion	2 (5%)
*Occipital nerve stimulator implant n (%)*	
Yes	6 (15%)
No	34 (85%)

IQR, Interquartile range; SD, standard deviation.

**Figure 1:**
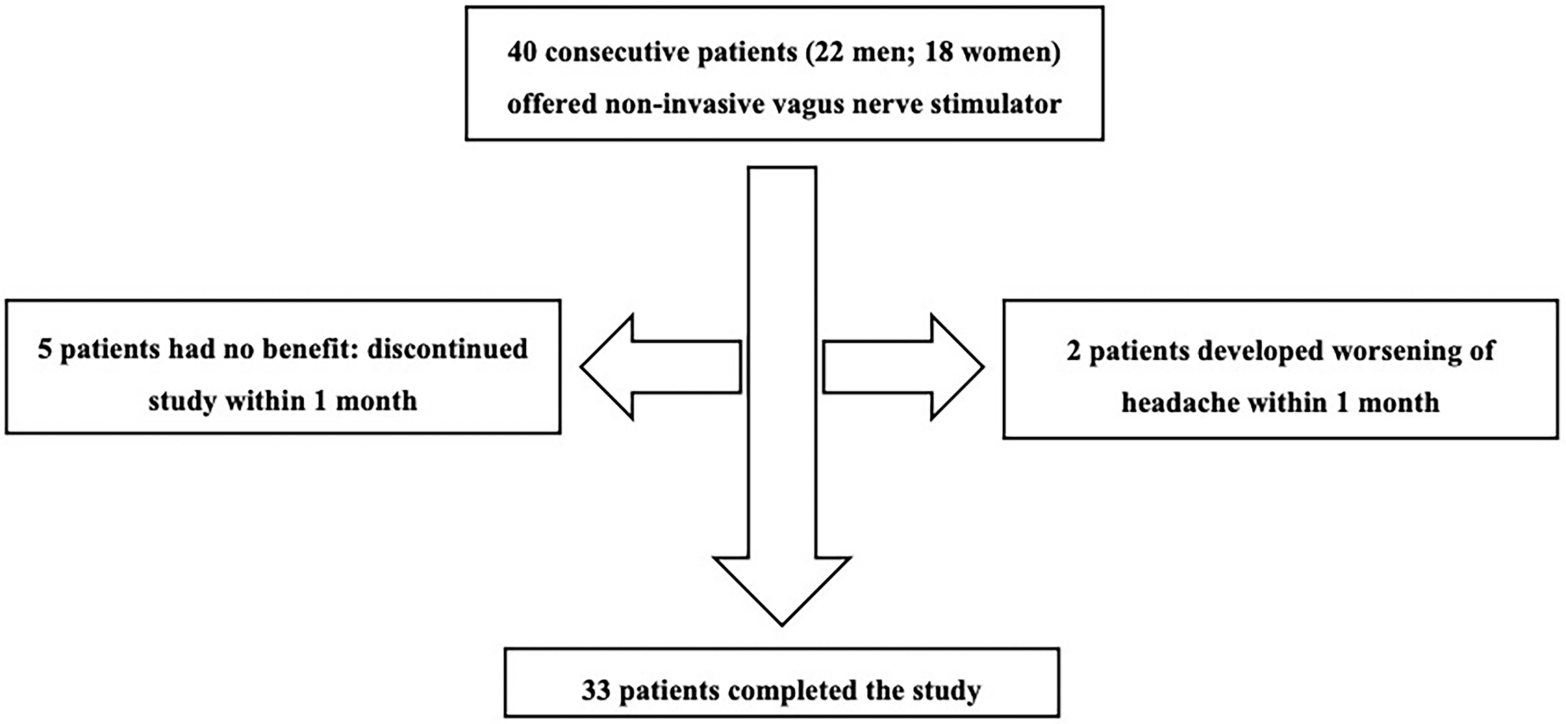
Flow of patients through the study.

Figure 1 shows the patient flow through the study. Five patients found no benefit and two developed worsening headaches. These patients discontinued use of the device within 1 month and did not complete diaries after baseline. Thus 7 patients had missing headache diary data. These patients were considered non-responders and included in analyses using last observation carried forward from baseline.

Review of the prospective headache diaries showed that 43% of patients (17/40) achieved a reduction in mean monthly attack frequency of 50% or more at 3 months. Two patients achieved complete resolution of their cluster attacks by 3 months that continued to final follow up. There was a significant improvement in monthly attack frequency at 3 months compared to baseline (44.7; 95% CI 25.1 to 64.3; *p* < 0.001). This corresponded to a mean reduction in monthly headache frequency of 36% (95% CI 25 to 47). There was a significant reduction in average VRS severity at 3 months compared to baseline (1.2; 95% CI 0.5 to 1.9; *p* = 0.001). There was also a reduction in the duration of attacks of 10.5 min (95% CI 0.2 to 20.9; *p* = 0.046), but this did not reach significance after correcting for multiple comparisons. There was a reduction in the cohort’s HIT-6 scores of 3.3 points (95% CI 0.9 to 5.7; *p* = 0.009), which did not reach statistical significance after Bonferroni correction. Table 2 gives an overview of outcomes at baseline and 3 months.

**Table 2:** Non-invasive vagus nerve stimulation using the gammaCore^®^ device in chronic cluster headache.

	BASELINE Mean (SD) Median (IQR)	3 months Mean (SD) Median (IQR)	Mean difference (95% CI)	*p* value^*^
Frequency (attacks/month)	124 (±67) 107 (65–174)	79 (±63) 65 (33–120)	−44.7 (−25.1 to −64.3)	<0.001
Severity (VRS)	8 (±2) 8 (7–10)	7 (±3) 7 (5–9)	−1.2 (−0.2 to −1.9)	0.001
Duration (minutes)	86 (±58) 68 (49–103)	75 (±64) 63 (35–100)	−10.5 (−0.15 to −20.9)	0.046
HIT-6	65 (±7) 65 (62–71)	62 (±10) 64 (58–67)	−3.3 (−0.9 to −5.7)	0.009
Triptan use (4 weeks)	32 (±36) 21 (0–56)	23 (±34) 7 (0–42)	−9.0 (−1.2 to −16.8)	0.024
Oxygen use (4 weeks)	46 (±62) 16 (0–82)	32 (±52) 8 (0–55)	−13.5 (0.1 to −27.0)	0.051

* The threshold for statistical significance based on Bonferroni adjustment is 0.008 (i.e., 0.05/6); SD, standard deviation; CI, confidence interval; HIT-6, Headache impact test – 6; VRS, Verbal Rating Score (0 = no pain;10 = excruciating pain).

Though there was a reduction in subcutaneous sumatriptan use at 3 months compared to baseline of 9.0 injections/month (95% CI 1.2 to 16.8; *p* = 0.024), this did not reach statistical significance after correcting for multiple comparisons. There was no significant change in oxygen use at 3months (MD = -13.5; 95% CI -0.1 to 27.0; *p* = 0.051).

With regards to adverse events, two patients reported an area of redness around the stimulation site that lasted up to 30 min and one reported a transient aching sensation over the neck muscles for 15 min after each stimulation. No patients were affected by this or felt the need to stop using the device as a result. The six patients with occipital nerve stimulator implants were able to use the nVNS without any adverse effects. Three of these patients reported continued benefit with the gammaCore device at follow up of 12, 12 and 25 months, respectively.

### Meta-analysis

**Figure 2:**
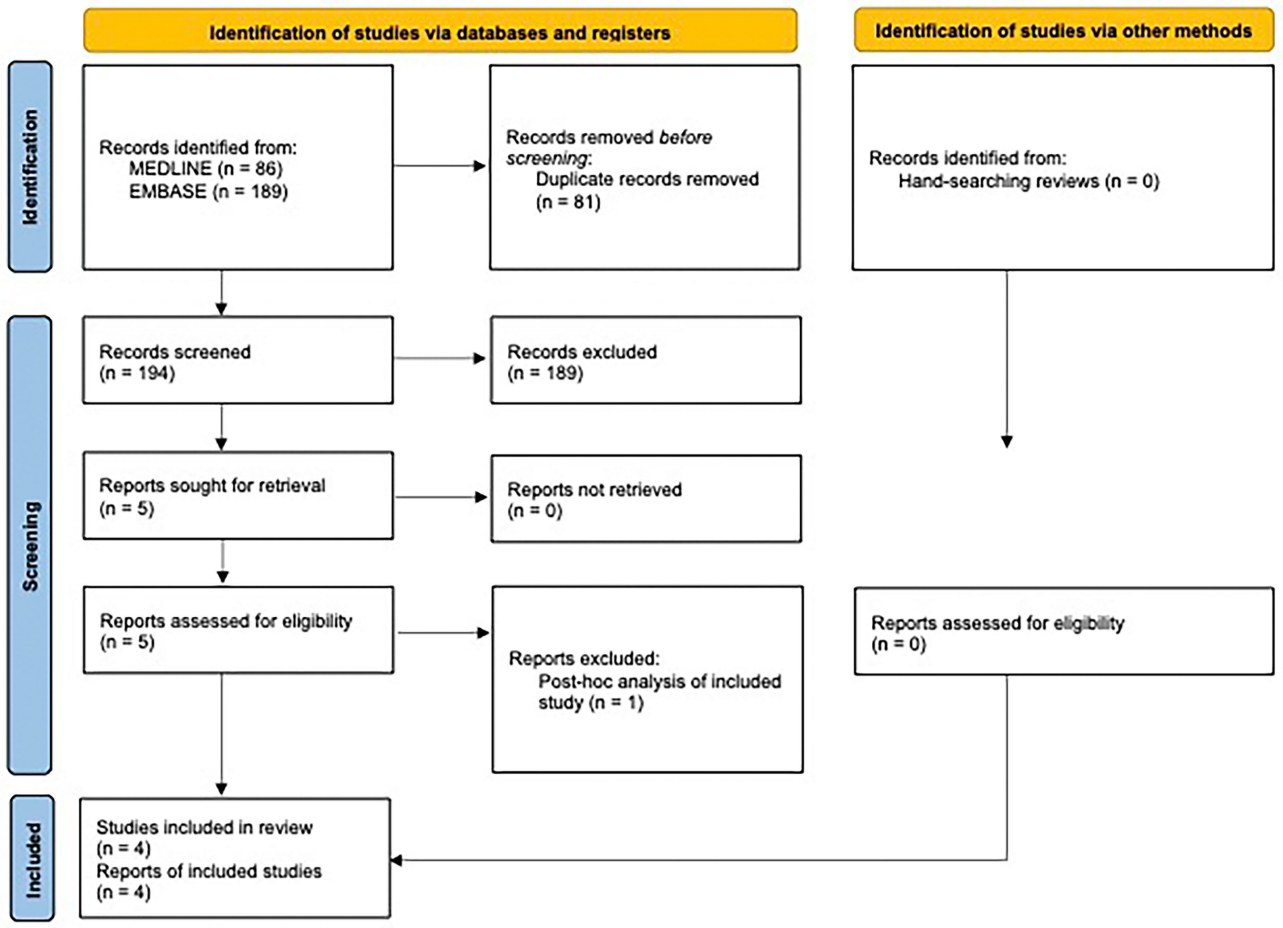
PRISMA flow diagram.

A complete overview of the study selection procedure is given in the PRISMA flowchart in Figure 2. A PRISMA 2020 Checklist is included in the Supplementary material.

The database search yielded 275 records, whereof 81 were duplicates. The abstracts of the remaining articles were screened, and 189 studies were excluded. Of these, 15 studies were excluded as their population did not meet our inclusion criteria. Eleven studies were excluded as the intervention used did not meet inclusion criteria. For example, studies of abortive rather than preventative use of nVNS, or use of an alternative neuromodulatory device or a drug trial. One hundred and sixty results were excluded as the study type did not fit our inclusion criteria; predominantly these were review articles (*n* = 119). Three studies were excluded as they did not report on relevant outcome measures.

Full text of the remaining five studies was screened, of which, 4 studies (10–12, 15), with a total of 97 patients, in addition to the present study, with 40 patients, met eligibility criteria and were included in the meta-analysis. One study was considered near-eligible, but was excluded as it was a post-hoc analysis of one of the included studies (11, 22). The reference lists of four key reviews were hand searched but did not reveal any additional relevant studies (23–26).

**Figure 3:**
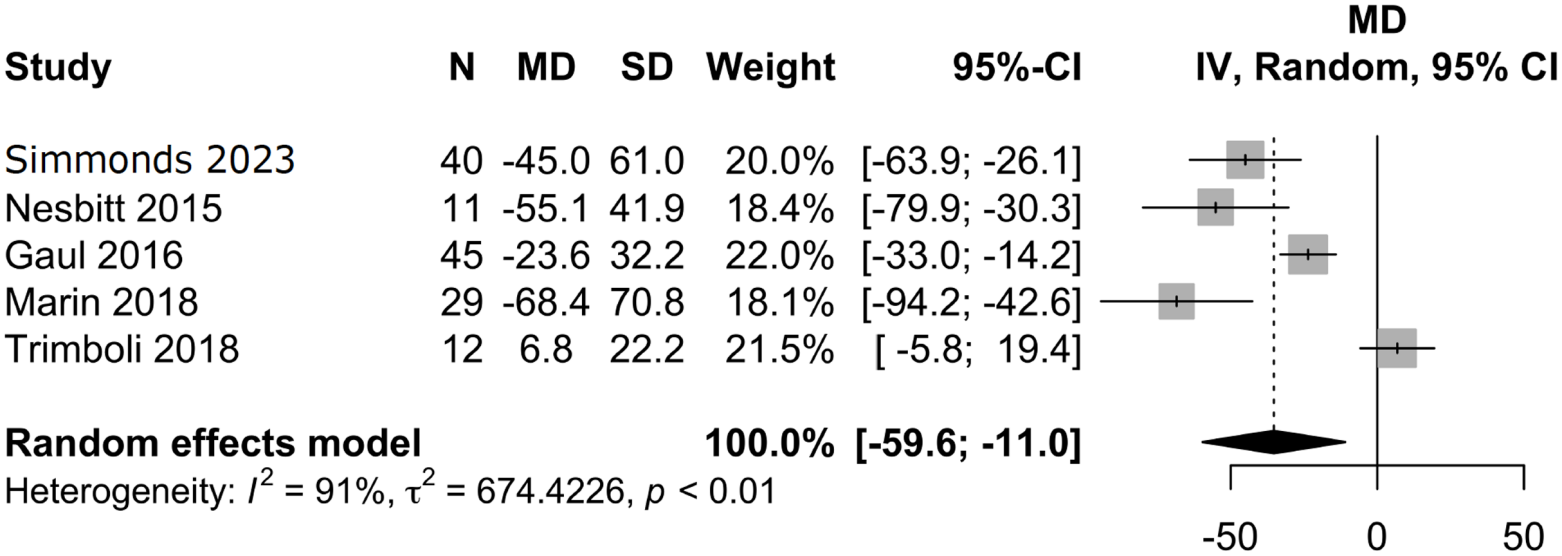
Meta-analysis of mean difference in monthly headache attack frequency between baseline and final follow-up.

**Figure 4:**
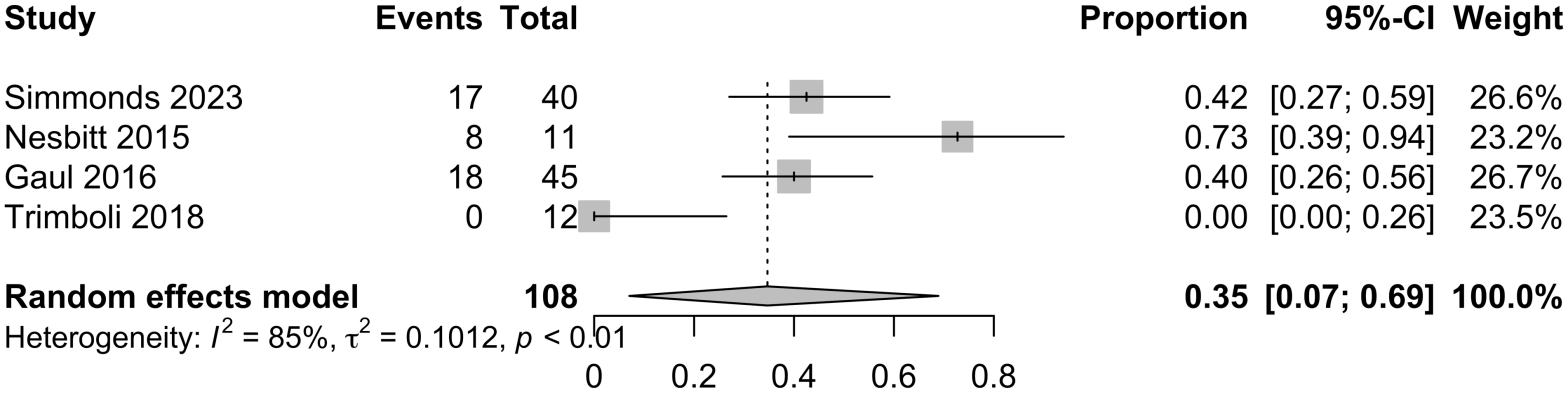
Meta-analysis of responder proportion.

Characteristics of the included studies and detailed risk of bias assessments are provided in the Supplementary material. The mean attack frequency at baseline was 105 (±22.7) attacks per month. nVNS as a preventative treatment for CCH, including refractory CCH, showed a mean difference in monthly headache attack frequency from baseline to final follow-up of −35.3 (95% CI −59.6 to 59 −11.0; Figure 3) in the analysis of all studies (10–12, 15), with a total of 137 patients. In this analysis 14/28 (50%) risk of bias assessments were deemed as moderate or without sufficient information, whilst the remaining were low. Analysis of three studies (10, 11, 15) with available responder outcome, in addition to the present study, (108 patients) showed a responder proportion of 0.35 (95% CI 0.07 to 0.69; *I*^2^ = 85%; Figure 4). In the responder proportion analysis 9/21 (43%) risk of bias assessments were deemed as moderate or without sufficient information to assess, whilst the remainder were low.

## Discussion

This is one of the largest single centre studies providing open-label evidence for the potential benefit of nVNS in patients with medically refractory CCH. Our results showed 17/40 patients achieving ≥50% reduction in headache frequency at final follow up. The response to nVNS complements other studies findings (10–12) along with the NICE clinical experts who identified a 25–50% response. Our meta-analysis further consolidates the notion that nVNS may be a treatment option for this difficult-to-treat group.

Activation of the trigemino-parasympathetic reflex is postulated to play a central role in the pathophysiology of CH. CH attacks are caused by activation of the trigeminal nerve and stimulation of the parasympathetic autonomic system resulting in the associated cranial autonomic symptoms (27). Evidence suggests that there is central modulation of the reflex by the hypothalamus (27, 28). Whether the hypothalamus drives the attacks or whether it is involved in putting the trigeminal autonomic reflex into a permissive state which facilitates the initiation of the attacks remains uncertain (27).

The vagus nerve has multiple associations with areas involved in pain regulation including the spinal trigeminal nucleus. Earlier studies suggested the acute effect of vagus nerve stimulation is facilitated through direct inhibition of afferent nerves to the caudal trigeminal nucleus (29). Recent neuroimaging studies have demonstrated the inhibition of areas identified as part of the pain matrix, including the limbic structures and the brainstem (30). Vagus nerve stimulation may reduce glutamate concentrations within the trigeminal nucleus caudalis therefore reversing central sensitisation in chronic headache (31). The modulation of the trigeminal autonomic reflex by nVNS was further investigated by Moeller et al. (32) in a study where lacrimation in healthy volunteers was significantly reduced in patients who received cervical nVNS compared to no stimulation or sham stimulation. They concluded that the reduction in lacrimation occurred either due to top-down modulation *via* the hypothalamus or through the direct bilateral inhibitory effect on the parasympathetic function within the trigeminal autonomic reflex arc.

The clinical evidence for preventative use of nVNS in CCH comprises the four studies included in our meta-analysis, each with methodological and clinical implications that supplement our findings.

Firstly, an audit by Nesbitt et al. including 19 CH patients (11 CCH and 7 refractory CCH), identified a self-reported overall improvement among 48% of patients after 1 year (11). They further concluded that prophylactic use of the gammaCore resulted in a substantial reduction in estimated mean attack frequency. However, limitations identified in this study include changes in preventative medication use alongside the nVNS and the small sample size. We kept preventative medication unchanged throughout our study period and included a larger population.

Secondly, a larger multicentre, prospective, open-labelled randomised study (PREVA) investigated the prevention and acute treatment of CCH (11). The study compared 48 patients receiving adjunctive prophylactic nVNS plus standard care to 49 patients who received standard care alone for 4 weeks, followed by a four-week extension of standard care and nVNS. Those receiving nVNS during the randomised phase had 3.9 less attacks a week (*p* = 0.002) than the controls. Those who continued using nVNS and those who added nVNS during the extension period also reported an additional reduction in attack frequency. They concluded that nVNS is beneficial as a preventative in chronic cluster headaches and identified additional improvement with continued use. The sustained effect was confirmed in a *post hoc* analysis (22). In our study, patients responding at 3 months continued to get benefit at final follow up, supporting the sustained effect from the PREVA reports.

Thirdly, Trimboli et al. investigated the preventative and acute effects on nVNS in patients with chronic primary headaches, including 12 with refractory CCH (15).They identified only one CCH patient who benefitted from nVNS and concluded that nVNS may be an ineffective acute or preventative treatment in refractory chronic primary headaches. The difference seen between this study and other studies may relate to the degree of patient contact and nVNS adherence. Patients in Trimboli’s study were seen at 3 months with additional phone contact if needed, possibly limiting adherence. On the other hand, the PREVA study reported 80% adherence by 64.4% of patients (11), and in our study 83% of patients had 100% adherence. The high adherence rate may relate to ensuring the appropriate use of the device, helped by having regular follow up and close monitoring of patients in the early stages of treatment. It is also possible that closer follow up with patients may have led to other potential unmeasured benefits outside of nVNS use.

Finally, a recent multicentre audit by Marin et al. (12) looked retrospectively at 30 patients with CH (29 chronic; 1 episodic). The mean evaluation period was 7.6 months, with 16 CCH patients exclusively using nVNS as a preventative measure. At the end of the study, three patients had had no further attacks. They found a significant decrease in attack frequency, duration and severity in patients previously not responding or intolerant of multiple preventatives, contradicting the results of the Trimboli study (15). The mean duration of nVNS use in our study was 9 months with two patients having no further attacks by the end of the study. However, Marin et al. recognised an inclusion bias with patients known to respond to adjunctive nVNS. Our patients had never received nVNS and therefore responses are more representative of the CCH population. In addition, patients in the Marin et al. audit used nVNS as both acute and preventative treatment, which may have synergistic effect. The results seen in our study were from preventative nVNS use alone.

The meta-analysis presented in this paper are in line with the findings from the present study and several of the above-mentioned studies. However, the meta-analysis should be interpreted with caution, and rather serves as an indication of the treatment’s efficacy rather than being high-level evidence. There are several potential sources of bias. Firstly, the analyses were conducted as single-arm meta-analyses, not providing the robustness usually seen with meta-analysis of comparative randomised trials. Secondly, all included studies were judged to at least some degree of moderate risk of bias, which is inevitable in open-label observational designs. Finally, there was heterogeneity in both statistical and clinical variables across the studies, including heterogeneity of the populations with a mixture of refractory and non-refractory patients, which further decrease the confidence in the estimates. Nevertheless, the majority of patients in the meta-analysis were constituted of refractory CCH patients, and our estimates appear to be among the most comprehensive in the published literature.

Despite the reductions in attack frequency, there are some uncertainties around the disability impact of nVNS. The lack of significant improvement of the HIT-6, despite decrease in attack frequency does not necessarily indicate lack of efficacy of the nVNS. This discrepancy could be explained by the scoring system not being validated for cluster headaches and therefore its sensitivity for detecting symptom improvements may be low. This highlights the need for a validated quality of life measure for trigeminal autonomic cephalalgias.

Our observational study is limited by the lack of a placebo control. It is difficult to design a robust sham neurostimulation device or stimulation programme whilst maintain blinding as participants are able to feel the stimulation. There are several strengths to our study including prolonged follow-up; the large number of refractory CCH within a single centre; prospective data collection; the “real life” nature of the data and the closeness of follow up with easy access to specialists; and high adherence rates. A further potential confounder is the close monitoring patients received, which may, in itself, have a therapeutic role. The meta-analysis is limited by the inherent shortcomings of single-arm meta-analysis. Additionally, all studies relied on patients’ recollection and recording of headache data in a diary, which can lead to over or under reporting, and missing data points. This could be minimised with prospective data collection, frequent follow up and user-friendly paper or electronic diaries, but remains an inherent challenge when monitoring chronic pain conditions.

Our study suggests non-invasive neuromodulation techniques with the gammaCore may be a beneficial preventative measure in patients with CCH for whom current treatments are not tolerated or ineffective. We demonstrated a significant reduction in cluster headache frequency and severity. Furthermore, the treatment has favorable adverse effect profiles making it a suitable alternative for patients’ intolerant of other treatments. This prospective study highlights the benefits of close monitoring for patients treated with nVNS including ensuring optimum use of the device, regular reviews at 1 month and three-monthly thereafter and easy access to headache specialist teams. Patients should be encouraged to continue nVNS for at least 3 months to accurately establish any treatment effect. These promising results alongside other open-label data identifies the need for a high-quality, double-blinded, randomised control study investigating the role of gammaCore in the preventative treatment of CCH.

The approval of non-invasive stimulators, such as the vagus nerve stimulator, by regulatory bodies has occurred despite a paucity of randomised controlled trials supporting their efficacy. We posit that this threshold for approval is too low and recommend that regulatory authorities re-evaluate their policies. Whilst we have observed a favorable effect of vagus nerve stimulation on chronic cluster headache, as have others through single arm meta-analyses, it is important to note that this may be primarily due to a placebo response. In fact, randomised controlled studies (33, 34) investigating preventive therapies for cluster headache have reported a substantial placebo response, similar to the response rates seen in open-label studies evaluating vagus nerve stimulation for chronic cluster headache. Therefore, it is imperative that the manufacturer and the headache community execute rigorous randomised controlled trials prior to the adoption of this device as standard therapy.

## Data availability statement

The raw data supporting the conclusions of this article will be made available by the authors, without undue reservation.

## Ethics statement

Ethics board approval for the collection and publication of data was granted by Northwick Park Hospital Research Ethics Committee, London, UK (reference number: 11/LO/1709). The patients/participants provided their written informed consent to participate in this study.

## Author contributions

LS: drafting and revision of manuscript. SK: analysis and interpretation of data, drafting, and revision of manuscript. SL: data collection, analysis and interpretation of data, and revision of manuscript. AS: analysis and interpretation of data and revision of manuscript. SC: interpretation of data and revision of manuscript. ET: study concept, interpretation of data, and revision of manuscript. MM: study concept, recruitment of subjects, interpretation of data, and manuscript revision. All authors contributed to the article and approved the submitted version.

## Funding

ElectroCore part-funded the study. The funder had no involvement in the study design, data collection, analysis or publication of the study. The funder was not involved in the meta-analysis.

## Conflict of interest

SL has received payment for attending advisory meetings and development of presentation from Allergan Novartis, Eli Lilly and TEVA. AS is co-founder of Nordic Brain Tech, a company developing a non-pharmacological biofeedback treatment for migraine and holds a pending patent application relating to the company’s product. ET is co-founder and shareholder of Nordic Brain Tech AS and Palion Medical AS. He serves on advisory board for Eli Lilly and Novartis and TEVA. He has received speaker honoraria from Eli Lilly, Novartis, Allergan and TEVA. MM serves on the advisory board for Abbott, Allergan, Eli Lilly, Medtronic, Novartis, TEVA; has received payment for the development of educational presentations from Allergan, electroCore, Eli Lilly, Medtronic, Novartis, and TEVA; and, has received research grants from Abbott, electroCore and Medtronic.

The remaining authors declare that the research was conducted in the absence of any commercial or financial relationships that could be construed as a potential conflict of interest.

## Publisher’s note

All claims expressed in this article are solely those of the authors and do not necessarily represent those of their affiliated organizations, or those of the publisher, the editors and the reviewers. Any product that may be evaluated in this article, or claim that may be made by its manufacturer, is not guaranteed or endorsed by the publisher.

## Supplementary material

The Supplementary material for this article can be found online at:

https://www.frontiersin.org/articles/10.3389/fneur.2023.1100426/full#supplementary-material

## Abbreviations

CH: Cluster headache
nVNS: Non-invasive vagus nerve stimulation
CCH: Chronic CH
NICE: National Institute for Health and Care Excellence
HIT-6: Headache Impact Test-6
VRS: Verbal rating scale
MD: Mean differences
CI: Confidence intervals
