# Supplementary material for: An open-label observational study and meta-analysis of non-invasive vagus nerve stimulation in medically refractory chronic cluster headache
Source: Front Neurol. 2023 Mar 30;14:1100426. doi: 10.3389/fneur.2023.1100426 (PMC10098146; doi:10.3389/fneur.2023.1100426)
Supplement: Supplementary file 1 [file Data_Sheet_1.docx]

**Supplementary material.** Characteristic and risk of bias of studies included in meta-analysis.

| **GAUL 2016** | | |
| --- | --- | --- |
| **Methods** | Prospective, multicentre, open-label, randomised, controlled, two-armed, parallel-group study. | |
| **Participants** | Chronic CH according to ICHD-3. Discussion states that patients were refractory to medical treatments. 97 patients randomized, 92 completed randomization phase. | |
| **Interventions** | Standard of care plus nVNS (n=48) versus standard of care alone (n=49). Three consecutive 2-minutes stimulation doses two times a day. Acute treatment as up to three consecutive stimulations to abort attacks | |
| **Outcomes** | Mean change in weekly attack frequency; responder proportion (defined as > 50% reduction in headache frequency); abortive medication use; duration and intensity of CH attacks that were treated with nVNS; quality of life assessment with EQ-5D-3L and HIT-6. | |
| *Risk of bias* | | |
| **Category** | **Risk** | **Reason** |
| Pre-intervention confounding | ‘low’ | Confounding unlikely due to randomized design and even distribution of baseline characteristics. |
| Pre-intervention selection bias | ‘low’ | Randomized design. |
| Bias in classification of intervention | ‘low’ | Misclassification unlikely due to prospective design. |
| Bias due to deviation from intended interventions | ‘low’ | An intention-to-treat analysis was carried out. |
| Bias due to missing data | ‘low’ | Missing data accounted for. |
| Bias in measurement of outcome | ‘moderate’ | Investigators and outcome assessors were aware of treatment allocation. |
| Bias in selection of the reported result | ‘low’ | Prespecified outcomes reported. |
| **MARIN 2018** | | |
| **Methods** | Retrospective audit of real-world clinical data. | |
| **Participants** | 29 chronic CH and 1 episodic CH patients. All had failed 3 or more preventative treatments. | |
| **Interventions** | nVNS as preventative treatment, acute treatment or both in addition to standard of care. Dosing based on established stimulation paradigm and titrated to achieve maximum benefit. | |
| **Outcomes** | Mean weekly decrease in attack frequency from before to after treatment (follow-up time range 1.7 to 13.2 months); responder proportion not reported and not calculatable from reported data. | |
| *Risk of bias* | | |
| **Category** | **Risk** | **Reason** |
| Pre-intervention confounding | ‘no information’ | - |
| Pre-intervention selection bias | ‘moderate’ | Unclear which patients were offered nVNS. |
| Bias in classification of intervention | ‘low’ | Interventions likely not misclassified as they were identified from patient records. |
| Bias due to deviation from intended interventions | N/A | No comparator group. |
| Bias due to missing data | ‘moderate’ | Patients removed from analyses in case of missing data. |
| Bias in measurement of outcome | ‘moderate’ | Outcome assessors were aware of intervention and retrospective review of outcome could have influenced the measure. |
| Bias in selection of the reported result | ‘low’ | Prespecified outcomes reported. |
| **NESBITT 2015** | | |
| **Methods** | Open-label observational cohort study. | |
| **Participants** | Nineteen patients with active episodic or chronic CH according to “current classification criteria” attending 1 of 2 tertiary headache clinics. Of these 11 were chronic cluster headache patients and 7/11 were considered treatment refractory. | |
| **Interventions** | nVNS as an acute and preventative treatment. Up to three consecutive doses as acute treatment, and 2-3 consecutive doses in the morning and afternoon as preventative treatment. | |
| **Outcomes** | Patients subjective perception of overall benefit; 24-hour attack frequency; bout duration; change in acute medication; percentage of attacks aborted, and proportion of aborted attacks terminated within 15 minutes. Outcomes were recorded on a headache diary. Responders (defined as >50% reduction in attack frequency was calculated in-house) from data provided in the report. | |
| *Risk of bias* | | |
| **Category** | **Risk** | **Reason** |
| Pre-intervention confounding | ‘moderate’ | Unclear how patients assigned to the intervention. |
| Pre-intervention selection bias | ‘moderate’ | Unclear how patients assigned to the intervention. |
| Bias in classification of intervention | ‘low’ | Interventions likely not misclassified as they were identified from patient records. |
| Bias due to deviation from intended interventions | ‘moderate’ | Four patients had changes to baseline treatment during the intervention period. |
| Bias due to missing data | ‘moderate’ | Patients without data excluded from analyses. No further description of handling of missing data. |
| Bias in measurement of outcome | ‘moderate’ | Outcome assessors were aware of intervention and retrospective review of outcome could have influenced the measure. |
| Bias in selection of the reported result | ‘low’ | Prespecified outcomes reported. |
| **TRIMBOLI 2018** | | |
| **Methods** | Open-label prospective clinical audit | |
| **Participants** | 41 chronic primary headache patients, whereof 12 refractory chronic cluster headache patients according to IHS criteria. | |
| **Interventions** | nVNS in a preventative stimulation paradigm with two consecutive doses three times a day. In addition, acute treatment with up to three consecutive doses to abort attacks. | |
| **Outcomes** | Responders defined as 30% or greater reduction in headache days/episodes. Frequency, intensity, and duration of headache episodes; days with acute headache medication consumption; change in headache-related disability; patients’ subjective satisfaction with the treatment. | |
| *Risk of bias* | | |
| **Category** | **Risk** | **Reason** |
| Pre-intervention confounding | ‘No information’ | - |
| Pre-intervention selection bias | ‘low’ | Consecutive patients meeting diagnostic criteria were included. |
| Bias in classification of intervention | ‘low’ | Interventions likely not misclassified as were administered prospectively. |
| Bias due to deviation from intended interventions | N/A | No comparator group. |
| Bias due to missing data | ‘low’ | No missing data for outcome of interest. |
| Bias in measurement of outcome | ‘moderate’ | Outcome assessors were aware of intervention and retrospective review of outcome could have influenced the measure. |
| Bias in selection of the reported result | ‘low’ | Prespecified outcomes reported. |

nVNS=non-invasive vagus nerve stimulation; CH=cluster headache; IHS=international headache society
